# Genetically Modifying the Insect Gut Microbiota to Control Chagas Disease Vectors through Systemic RNAi

**DOI:** 10.1371/journal.pntd.0003358

**Published:** 2015-02-12

**Authors:** Mabel L. Taracena, Pedro L. Oliveira, Olivia Almendares, Claudia Umaña, Carl Lowenberger, Ellen M. Dotson, Gabriela O. Paiva-Silva, Pamela M. Pennington

**Affiliations:** 1 Instituto de Bioquímica Médica Leopoldo de Meis, Programa de Biologia Molecular e Biotecnologia, Universidade Federal do Rio de Janeiro, CCS, Ilha do Fundão, Rio de Janeiro, Brasil; 2 eCentro de Estudios en Salud. Universidad del Valle de Guatemala, Guatemala City, Guatemala; 3 Instituto Nacional de Ciência e Tecnologia em Entomologia Molecular (INCT-EM), Brasil; 4 Centers for Disease Control and Prevention, Division of Parasitic Diseases and Malaria, Atlanta, Georgia, United States of America; 5 Department of Biological Sciences, Simon Fraser University, Burnaby, British Columbia, Canada; Universidad Autónoma de Yucatán, MEXICO

## Abstract

Technologies based on RNA interference may be used for insect control. Sustainable strategies are needed to control vectors of Chagas disease such as *Rhodnius prolixus*. The insect microbiota can be modified to deliver molecules to the gut. Here, *Escherichia coli* HT115(DE3) expressing dsRNA for the Rhodnius heme-binding protein (RHBP) and for catalase (CAT) were fed to nymphs and adult triatomine stages. RHBP is an egg protein and CAT is an antioxidant enzyme expressed in all tissues by all developmental stages. The RNA interference effect was systemic and temporal. Concentrations of *E. coli* HT115(DE3) above 3.35 × 10^7^ CFU/mL produced a significant RHBP and CAT gene knockdown in nymphs and adults. RHBP expression in the fat body was reduced by 99% three days after feeding, returning to normal levels 10 days after feeding. CAT expression was reduced by 99% and 96% in the ovary and the posterior midgut, respectively, five days after ingestion. Mortality rates increased by 24-30% in first instars fed RHBP and CAT bacteria. Molting rates were reduced by 100% in first instars and 80% in third instars fed bacteria producing RHBP or CAT dsRNA. Oviposition was reduced by 43% (RHBP) and 84% (CAT). Embryogenesis was arrested in 16% (RHBP) and 20% (CAT) of laid eggs. Feeding females 10^5^ CFU/mL of the natural symbiont, *Rhodococcus rhodnii*, transformed to express RHBP-specific hairpin RNA reduced RHBP expression by 89% and reduced oviposition. Modifying the insect microbiota to induce systemic RNAi in *R. prolixus* may result in a paratransgenic strategy for sustainable vector control.

## Author Summary

*Rhodnius prolixus* is an important vector of Chagas disease. The development of insecticide resistance in triatomines has raised the need for new control methods. We propose, as a proof-of-concept, the use of symbiotic bacteria expressing dsRNA in a paratransgenic approach to control vector-borne disease. We first show that ingestion of *E. coli*, producing long dsRNA specific for *R. prolixus* genes, can produce systemic RNAi in this insect. By targeting genes with antioxidant function (RHBP and catalase), we show that RNAi effects on nymphs and adult females are systemic and temporal, affecting development and fecundity. Finally, we show that the natural vector symbiont, *R. rhodnii*, also can be modified to induce systemic RNA interference. The *E. coli* system can serve to screen potential targets for development of a symbiont-based vector control product that then can be transferred to *R. rhodnii*.

## Introduction

Oral delivery of dsRNA has been proposed as a method to control insect pests such as termites, fruit flies, flour beetles, pea aphids, tobacco hornworms and honeybees, among others [1–7]. These studies have shown variable efficacy of oral-mediated delivery, suggesting that each species and gene may behave differently. Paper impregnated with dsRNA was used effectively to silence genes in termites [7]. Plants coated with dsRNA, or genetically modified to express dsRNA specific for insect genes, also have been tested for the control of various plant pests [8], and bacteria have been used to deliver dsRNA in lepidopterans [9]. Studies on mosquito species that transmit diseases to humans showed effective oral delivery through exposure to dsRNA in solution [10] or stabilized in chitosan [11]. These studies suggest that oral delivery of dsRNA may provide a method to control disease vectors.

*Rhodnius prolixus* is an efficient vector of *Trypanosoma cruzi*, the etiological agent of Chagas disease, and is primarily controlled by domiciliary insecticide applications. Even though residual insecticide treatment inside houses is effective in decreasing the vector densities and minimizing the risk of infection [12], the sustainability of this method in the Central American and Andean regions is limited by the potential re-infestation by species from sylvatic habitats, the need for repeated applications of insecticides, and the potential development of resistance [13–15]. In 2002, the Tropical Disease Research Scientific and Technical Advisory Council and the World Health Assembly (resolution WHA 51.14) recommended that future research efforts focus on the development of control strategies for the local entomological conditions in these regions, where sylvatic vectors pose a serious threat to sustainability (http://www.who.int/tdr/diseases/chagas/strat_dir_chagas/en/index2.html#).

The use of genetically transformed insect symbiotic bacteria to block parasite transmission has been proposed as a sustainable control method for vector-borne diseases. The bacteria are modified to express an anti-parasite peptide to kill the parasite in the intestine of the vector [16–18]. Bacterial ingestion would allow introduction of transformed bacteria into a vector population [19]. Given that the vector is indirectly transformed through its symbionts, they are considered paratransgenic insects. Our group has worked for the past decade on the development of a paratransgenic model for transmission-blocking control for Chagas disease. The strategy is based on oral delivery of intestinal symbionts of *R. prolixus* in a paste-like formulation simulating triatomine feces [16, 19, 20]. *R. prolixus* harbors extracellular actinobacteria, *Rhodococcus rhodnii*, in the intestinal lumen [21]. The bacteria are shed in the feces and spread horizontally through the vector’s coprophagic habits [22]. Following a blood meal, *R. rhodnii* grows exponentially in the anterior midgut, the same compartment where *T. cruzi* establishes after entering the insect in an infected blood meal. Populations of *R. rhodnii* can reach 10^9^ CFU per 5^th^ instar nymph 10 days after the bloodmeal [23].

Coprophagous transmission and fast bacterial growth in the same location as the parasite allow oral transmission of transformed symbionts and production of antiparasitic molecules in the insect midgut. An adaptation of this strategy would be to transform the symbiotic bacteria to attack the vector’s physiology in addition to its vectorial competence. We propose that transformed gut symbiotic bacteria may be ingested by *R. prolixus* to produce dsRNA specific for genes important in insect development, fecundity and vectorial competence.

As a proof-of-concept, we used *Escherichia coli* [HT115(D3)][24] and *R. rhodnii* to deliver dsRNA specific for genes with antioxidant functions and involved in egg production and development: Rhodnius heme binding protein (RHBP) and catalase (CAT) [25–28]. Hematophagous insects are exposed to large amounts of free heme derived from the blood meal. Heme is a pro-oxidant molecule that stimulates lipid peroxidation by increasing reactive oxygen species (ROS) production [29]. Heme-RHBP is an egg protein that enters the developing oocytes through receptor-mediated endocytosis and is used as a heme source for embryogenesis [30–32]. It is secreted in all developmental stages by the fat body as a heme-bound protein and circulates in the hemolymph in apo and heme forms [33]. Catalase is a ubiquitous enzyme responsible for dismutation of hydrogen peroxide into molecular oxygen and water [34]. Catalase activity has been described in many tissues of *R. prolixus* [27] and plays a key role in the protection against oxidative damage [35]. Thus, RHBP and CAT physiologically protect cellular components against heme-induced oxidative damages. Knocking down RHBP expression affects the physiology and fecundity of females [30].

We proposed that oral delivery of bacteria expressing dsRNA for CAT or RHBP should affect the normal development of nymphs and female fecundity. Our results show that *E. coli* can express and deliver RHBP and CAT dsRNA into the midgut of *R. prolixus*, leading to the systemic spread of RNAi to the fat body, anterior and posterior midgut, and ovaries. Modified symbiotic *R. rhodnii* also were effective at inducing gene silencing with physiological effects. The potential application of this technology using bacterial symbionts for vector-borne disease control is discussed.

## Methods

### Insects

We used nymphs and adult stages of *R. prolixus* maintained at 28°C and 70% relative humidity. Conditions were maintained as described in S1 Text.

### Ethics statement

All animal care and experimental protocols were conducted following the guidelines of the institutional care and use committee (Committee for Evaluation of Animal Use for Research from the Federal University of Rio de Janeiro, CAUAP-UFRJ) and the NIH Guide for the Care and Use of Laboratory Animals (ISBN 0–309–05377–3). The protocols were approved by CAUAP-UFRJ under registry #IBQM001. Technicians dedicated to the animal facility at the Institute of Medical Biochemistry (UFRJ) carried out all aspects related to rabbit husbandry under strict guidelines to ensure careful and consistent handling of the animals.

### Plasmids and bacterial strains

Fragments from RHBP (375 bp) and CAT (453 bp) genes were cloned from *R. prolixus* cDNA into pGEM-T (Promega, Madison, Wisconsin, USA) plasmids to generate dsRNA, AINTEGUMENTA (ANT) gene from *Arabidopsis thaliana* was used as a control. Additionally, a RHBP and random nucleotide hairpin of ~100 bp were cloned into pBP2lac, an integration plasmid that integrates into the *R. rhodnii* chromosome (see S1 Text for details). All plasmids for dsRNA were purified and cloned into *E. coli* HT115(DE3) competent cells as described by Timmons [36] and plasmids with hairpins into *R. rhodnii* as described by Crampton et al [37]. PCR and transformation conditions are specified in SI text.

### Bacterial culture and feeding conditions

*E. coli* HT115(DE3) containing the pGEM-T plasmid with inserts for RHBP, CAT, and the ANT gene, and without plasmid was grown in LB media containing ampicillin and/or tetracycline. After induction, the bacterial cells were harvested by centrifugation and a pellet was resuspended in fresh rabbit blood for artificial feeding. Each group was fed separately on individual feeding chambers containing blood. Three different concentrations of *E. coli* were used: 2.24 × 10^7^ CFU/mL, 3.35 × 10^7^ CFU/mL, and 5.4 × 10^7^ CFU/mL. For the experiment with *R. rhodnii*, the bacteria containing the pBP2lac plasmid with inserts for RHBP and random nucleotides was grown in Brain Heart Infusion media containing kanamycin. For feeding, 4.8 × 10^5^ CFU *R. rhodnii*/mL of blood was used.

### Verification of knockdown at mRNA and protein level

The RNA from fat bodies, ovaries, posterior- and anterior- midguts of individual females was collected at different days post feeding. Gene expression was measured by cDNA synthesis and real time quantitative PCR (q-PCR). RNA from first and third stage nymphs was extracted from fat bodies and midgut. To evaluate apo-RHBP levels, hemolymph was collected from females. The binding of heme to RHBP was measured by progressively adding heme to 1 µl female hemolymph and recording the absorption spectra after each addition [33]. To evaluate CAT activity, anterior midguts from female adult insects were individually homogenized and used to measure CAT specific activity by spectrophotometry [27]. Midguts were also incubated with an oxidant-sensitive fluorophore dihydroethidium (DHE) and epifluorescence microscopy was performed. Experimental procedures are detailed in S1 Text.

### Phenotype description

Adult females were maintained in individual chambers during the oviposition cycle and eggs were collected daily [38]. Ovaries were dissected and photographed. Nymphs were also observed daily to track mortality and molting.

### Data analysis

ANOVA and Mann-Whitney tests were performed with the SPSS 17.0 software (IBM, Armonk, NY, USA) and Graph Pad Prisma (GraphPad Prism version 5.04 for Windows, GraphPad Software, La Jolla California USA, www.graphpad.com). All significant values have a p value less than 0.05.

### Accession numbers

Rhodnius heme-binding protein GenBank: AF493801.1 Catalase Vector Base Transcript ID: RPRC007907_RA.

## Results

### Establishment of feeding conditions in females with RHBP construct

We amplified, cloned, sequenced, and expressed RHBP and CAT dsRNA in *E. coli*, a sequence from *A. thaliana* was used as a control gene. Production of dsRNA after IPTG induction was confirmed by purification of dsRNA of each specific bacterial culture. Gel electrophoresis showed the expected products of approximately 400 bp (S1A Fig.). Based on spectrophotometric analysis of triplicate extractions, each mL of culture at an OD_600_ 0.8–1 had a median (interquartile range) of 1,542 (1,068–2,751) ng dsRNA for the RHBP *E. coli* strain and 1,944 (521–3,777) ng for the ANT *E. coli* strain. Insects were fed blood containing *E. coli* that expressed the dsRNA gene fragments, bacteria without plasmid, or blood free of bacteria. For the selection of appropriate concentrations of bacteria for the feeding, an “R” value was used to consider the amount of blood ingested in relation to the initial weight of each individual. Only females that engorged with >0.1 mL blood were included in the study. The amount of bacteria present in the blood showed no effect on R values even at the maximal bacterial concentration tested, 5.54 × 10^7^ CFU/mL (S2A Fig.). Daily weight loss due to diuresis and defecation was monitored five days after feeding (S2B Fig.). There was no difference in weight loss between the groups fed concentrations of *E. coli* of 2.24 × 10^7^, 3.35 × 10^7^and 5.4 × 10^7^CFU/mL. Individuals received at least 0.5–1.4 µg (5–14 ng dsRNA/µL blood) and 1–2.8 µg (10–28 ng dsRNA/µL blood) RHBP dsRNA at the lowest and highest bacterial concentrations, respectively.

### Verification of RHBP and CAT knockdown in adult females

**Figure 1:**
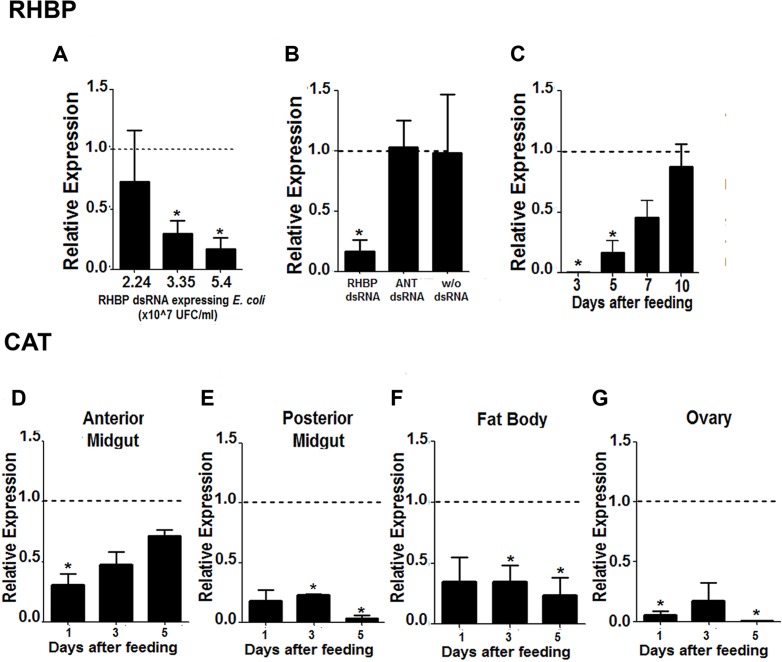
Dose- and time-dependent effect of RHBP knockdown, and tissue-dependent effect of CAT knock-down, in adult females. Females were fed *E. coli* expressing dsRNA. RHBP (A) Expression of RHBP relative to actin on day five after feeding with different amounts of bacteria expressing RHBP dsRNA as compared to insects fed sterile blood: 2.24 × 10^7^ CFU/ml of blood (n = 6), 3.35 × 10^7^ CFU/ml of blood (n = 12) and 5.4 × 10^7^ CFU/ml of blood (n = 8). (B) Expression of RHBP in insects fed 5.4 × 10^7^ CFU/mL blood using bacteria expressing RHBP dsRNA, ANT dsRNA, and without dsRNA. Asterisk indicates statistically different values (*T*-test, *P*< 0.05) between experimental groups exposed to bacteria with RHBP dsRNA (n = 6), bacteria with ANT dsRNA (n = 8), bacteria without dsRNA (n = 6), as compared to groups fed blood alone (n = 6). (C) Time-dependent relative expression of RHBP in insects fed 5.4 × 10^7^ CFU bacteria/ml blood ten days after feeding. Asterisk indicates statistically different values (*T*-test, *P*< 0.05) between each group and the group fed sterile blood. CAT (D-G) Tissue-specific silencing in females fed with 5.4 × 10^7^ CFU/mL *E. coli* HT115(DE3) expressing dsRNA CAT or blood alone. (D) Anterior midgut, (E) posterior midgut, (F) fat body and (G) ovaries from each individual were processed to measure expression of CAT, relative to controls. Bars represent SEM, three biological replicates (n = 3 per replicate). In all, asterisk indicates statistically different values as compared to controls fed with blood alone (*T*-test, *P*< 0.05).

The RHBP gene expression in the fat body was measured by q-PCR in individuals fed *E. coli* producing RHBP or ANT dsRNA, relative to individuals fed blood alone. In adults, a significant (*T*-test, *P*< 0.05) reduction in RHBP expression was observed after feeding *E. coli* expressing RHBP dsRNA at 3.35 × 10^7^ CFU/mL blood to 5.54 × 10^7^ CFU/mL blood (Fig. 1A). Experimental groups fed bacteria expressing ANT dsRNA or bacteria alone presented no significant difference (Two way ANOVA, p > 0.05) from the sterile blood control (Fig. 1B). Protein and mRNA expression increase over the first six days after feeding, dropping afterwards to constitutive levels [25, 33]. In females fed *E. coli* expressing RHBP dsRNA at 5.54 × 10^7^ CFU/mL blood, expression was significantly inhibited only on days three (99.6%) and five (85%) after feeding (*T*-test, *P*< 0.05) (Fig. 1C). Relative expression at day ten was significantly different from days three and five, suggesting temporal effects (ANOVA with Tukey post hoc test, *P*<0.05).

For CAT knockdown experiments, females were fed on the highest dose of *E. coli* as established with the RHBP construct. The knockdown response to bacteria expressing dsRNA for CAT was observed at least at one time point for all tissues tested (Fig. 1D-G). Gene expression levels were significantly reduced, relative to the controls, on day one in the anterior midgut (69%), days three and five in the posterior midgut (82 and 96% respectively), days three and five in the fat body (65 and 76% respectively), and days one and five in the ovary (94 and 96% respectively). CAT knockdown varied between 65–96% in all tissues and time points tested but showed no significant difference between tissues (ANOVA, p>0.05).

### Establishment of feeding conditions and verification of knockdown in nymphs

**Figure 2:**
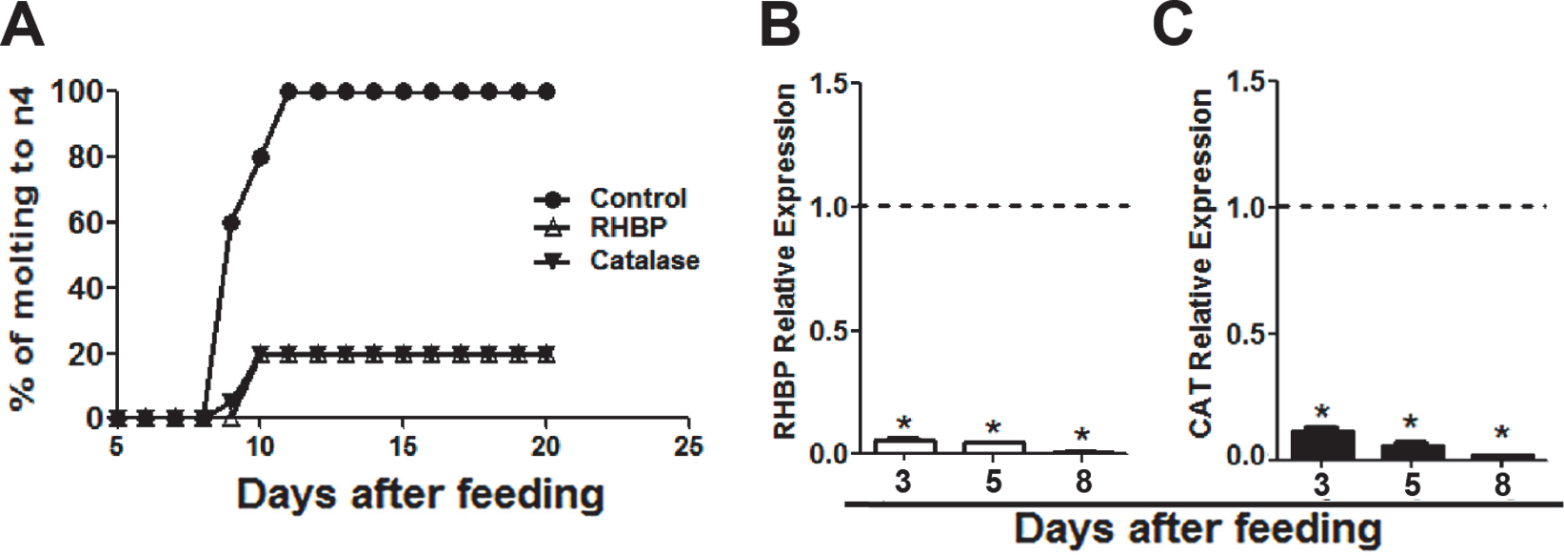
Inhibition of molting and reduction in transcription levels of RHBP and CAT in third instar nymphs. Nymphs were fed blood with *E. coli* producing RHBP and CAT dsRNA. (A) Reduction of molting in third instar nymphs fed bacteria producing RHBP or CAT dsRNA as compared with nymphs fed blood without bacteria and with bacteria expressing ANT dsRNA (two biological replicates, n = 10 each). (B) Relative expression of RHBP in third instar nymphs fed bacteria producing RHBP dsRNA (two biological replicates, n = 3 each). (C) Relative expression of CAT in midguts of third instar nymphs fed with bacteria producing RHBP dsRNA (two biological replicates, n = 3 each). Asterisk indicates statistically different values compared with the control fed blood alone (*T*-test, *P*< 0.05).

Optimal concentrations of *E. coli* for feeding first and third instar nymphs were determined using bacteria producing RHBP or CAT dsRNA. Third instars tolerated the same concentrations of bacteria as the adults without compromising viability (Table S1 in S1 Text). First instars showed 0–8% mortality in the control groups with a concentration of 2.5 × 10^7^ CFU/mL. First instars fed blood containing bacteria expressing RHBP dsRNA showed 30% mortality rates (Table S1 in S1 Text). The expression of RHBP and CAT in third instar nymphs was reduced by 99% on days three, five and eight after feeding (Fig. 2B and 2C). Molting was completely inhibited in surviving nymphs fed bacteria expressing RHBP or CAT dsRNA, as compared to nymphs fed blood alone (Table S1 in S1 Text). Only 20% of surviving third instars fed bacteria expressing RHBP or CAT dsRNA molted to the fourth instar, compared to 100% in the control groups (Fig. 2A). The results show that *E. coli* producing dsRNA for RHBP or CAT affect immature stage development and produce RHBP or CAT knockdown in third instars.

### Physiological effects of RHBP and CAT silencing in adult females

**Figure 3:**
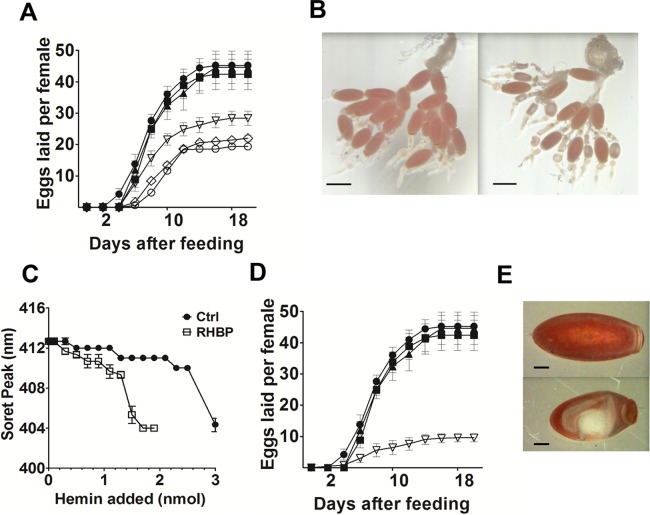
Physiological effects in adult females fed with *E. coli* expressing RHBP or CAT dsRNA. Reduction in oviposition, egg development and circulating apo-RHBP in females fed with *E. coli* producing RHBP dsRNA. (A) 20 day oviposition cycle. In black the three control groups (blood alone (circle), bacteria without dsRNA (square) and bacteria with ANT dsRNA (triangle)), in white the groups fed bacteria expressing RHBP dsRNA at three different concentrations 3.35 × 10^7^ CFU/mL blood (triangle), 4.02 × 10^7^ CFU/mL blood (rhombus) and 5.4 × 10^7^ CFU/ml blood (circle) (n = 5). Error bars represent SEM of three biological replicates. For one of the replicates, an additional control of uninduced RHBP bacteria was used, the insects (n = 8) showed no visible reduction from the normal number of eggs, ranging between 38–50 eggs/female. (B) Effects of the RHBP dsRNA in the ovaries of adult females of *R. prolixus* 10 days after feeding: left panel shows a control *R. prolixus* ovary. Right panel shows the effect of the inhibition of RHBP by feeding blood with bacteria expressing RHBP dsRNA. Bar = 1mm. (C) Reduction of apo-RHBP present in hemolymph seven days after feeding blood alone (ctrl) or 5.4 × 10^7^ CFU/mL blood bacteria expressing RHBP dsRNA (RHBP). Titration with hemin showed a significant reduction of the circulating apo-RHBP in knocked down insects (*T*-test, *P* = 0.0088). Bars represent SEM, three biological replicates, 1 representative female/replicate. As additional control of sample integrity, SDS-PAGE was performed to corroborate the protein profile. (D) Oviposition cycles in females fed blood alone (black circle), 5.4 × 10^7^ CFU/mL blood bacteria without dsRNA (black square), bacteria expressing ANT dsRNA (black triangle), and bacteria expressing CAT dsRNA (white triangle), (two biological replicates, n = 6 each). (E) The dehydration phenotype was observed in 20% of the eggs laid by females fed with bacteria expressing CAT dsRNA, at 5.4 × 10^7^ CFU/ml of blood. Bar = 0.2 mm.

Vitellogenesis in the telotrophic ovary begins within two days after the blood meal. After feeding, normal individuals initiate the first of three rounds of terminal oocyte development, consisting of approximately 14 eggs per round, for a total of approximately 42 eggs per 21 day oviposition cycle [38]. Each round produces terminal oocytes termed T0, T1 and T2. Females fed *E. coli* induced to express RHBP dsRNA showed a delay of one week in the first oviposition event, leading to a 43% reduction in the total number of eggs produced as compared to controls (Fig. 3A). During the oviposition cycle, the number of eggs oviposited by individuals of all groups was monitored individually and no significant difference was observed between the control groups (ANOVA, Tukey post hoc test, p>0.05). In the ovary of RHBP knockdown animals, the terminal oocyte development (T0) was inhibited at day ten post feeding (Fig. 3B). At the highest bacterial concentration, 5.54 × 10^7^ CFU/mL blood, the total reduction of eggs was 43%. Of the oviposited eggs, 16% (15 of 96) were white and non-viable, suggesting a reduction of heme-RHBP endocytosis in non-viable eggs [26]. The hemolymph protein profile was analyzed by titration of apo-RHBP at days three and seven after feeding. Comparing with the control groups fed blood alone or with *E. coli* expressing ANT dsRNA, a significant reduction of circulating apo-RHBP was observed in females fed *E. coli* expressing RHBP dsRNA at the seventh day post ingestion (*T*-test, *P*< 0.05) (Fig. 3C).

To evaluate if silencing had long term effects, a group of females was fed the bacteria during two consecutive gonotrophic cycles and the ovipositon was observed in both cycles. No difference in oviposition was observed between the first and the second feedings for the RHBP treatment (*T*-test, *P*>0.05) (S3 Fig.). There was no significant difference among the control groups (ANOVA, p>0.05).

**Figure 4:**
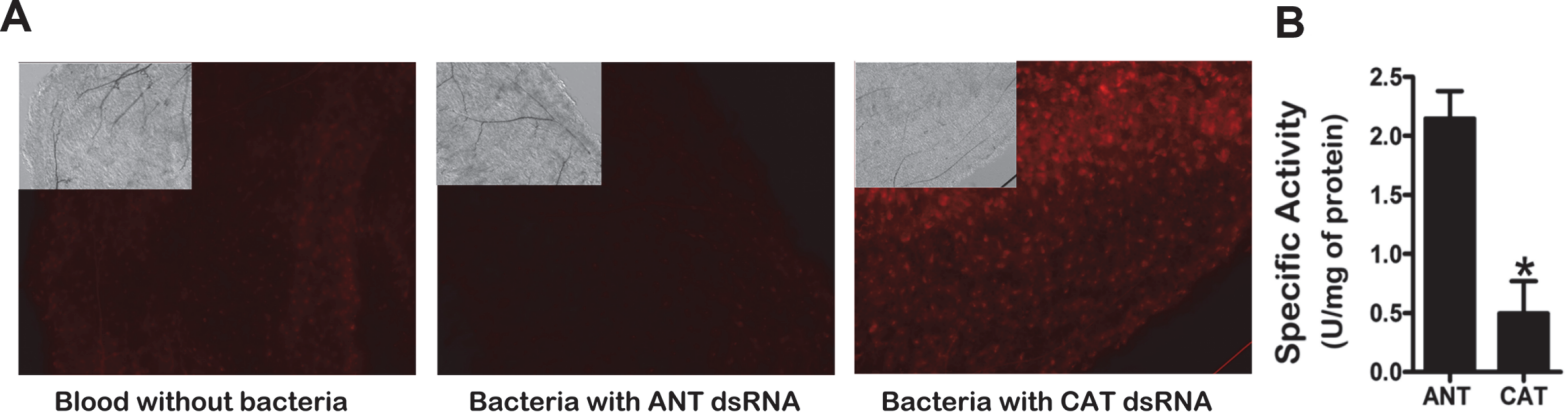
Reactive oxygen species and CAT specific activity in midguts of females fed with *E. coli* HT115(DE3) expressing CAT dsRNA. Females were fed blood alone, with *E. coli* HT115(DE3) expressing ANT dsRNA or CAT dsRNA, at 5.54 × 10^7^ CFU/mL blood. Midguts were dissected six days after feeding, incubated with dihydroethidium (DHE) and photographed under epifluorescence microscopy (Zeiss Observer.Z1 with Zeiss Axio Cam MrM using a filter set 10 (Exc 450–490 nm/ emission 515–565 nm)). Photographs show representative individuals from each group, inserts are differential interference contrast images. (B) Mean specific activity of CAT in insects fed *E. coli* HT115(DE3) expressing dsRNA CAT. Two biological replicates, n = 3 each. Error bars represent standard error of the mean. Asterisk indicates significant difference from control (*T*-test, *P*< 0.05).

When oviposition and egg development were evaluated in females fed *E. coli* expressing CAT dsRNA, the total number was reduced to 7–14 eggs per female, when normal females lay 39–54 eggs (Fig. 3D). Among a total of 9±3 eggs oviposited per female, 2±1 eggs (20%) presented a dehydrated phenotype (Fig. 3E) and were non-viable. To verify that the nonviability of the eggs was not related to lack of fertilization of the females, we performed a PCR for the Y chromosome using DNA extracted from normal eggs from control females and from dehydrated eggs from females knocked-down for CAT (S4 Fig.). All the samples were fertilized. ROS levels were observed and CAT specific activity was measured in the anterior midgut of females at day six after feeding the bacteria. The results showed a visible increase in midgut ROS levels upon CAT knockdown (Fig. 4A), together with a 76.89% (±16.79) reduction in CAT specific activity in relation to controls (Mann-Whitney, *P*<0.05) (Fig. 4B).

### RNAi effects in adult females after feeding with *R. rhodnii* expressing RHBP dsRNA hairpins

**Figure 5:**
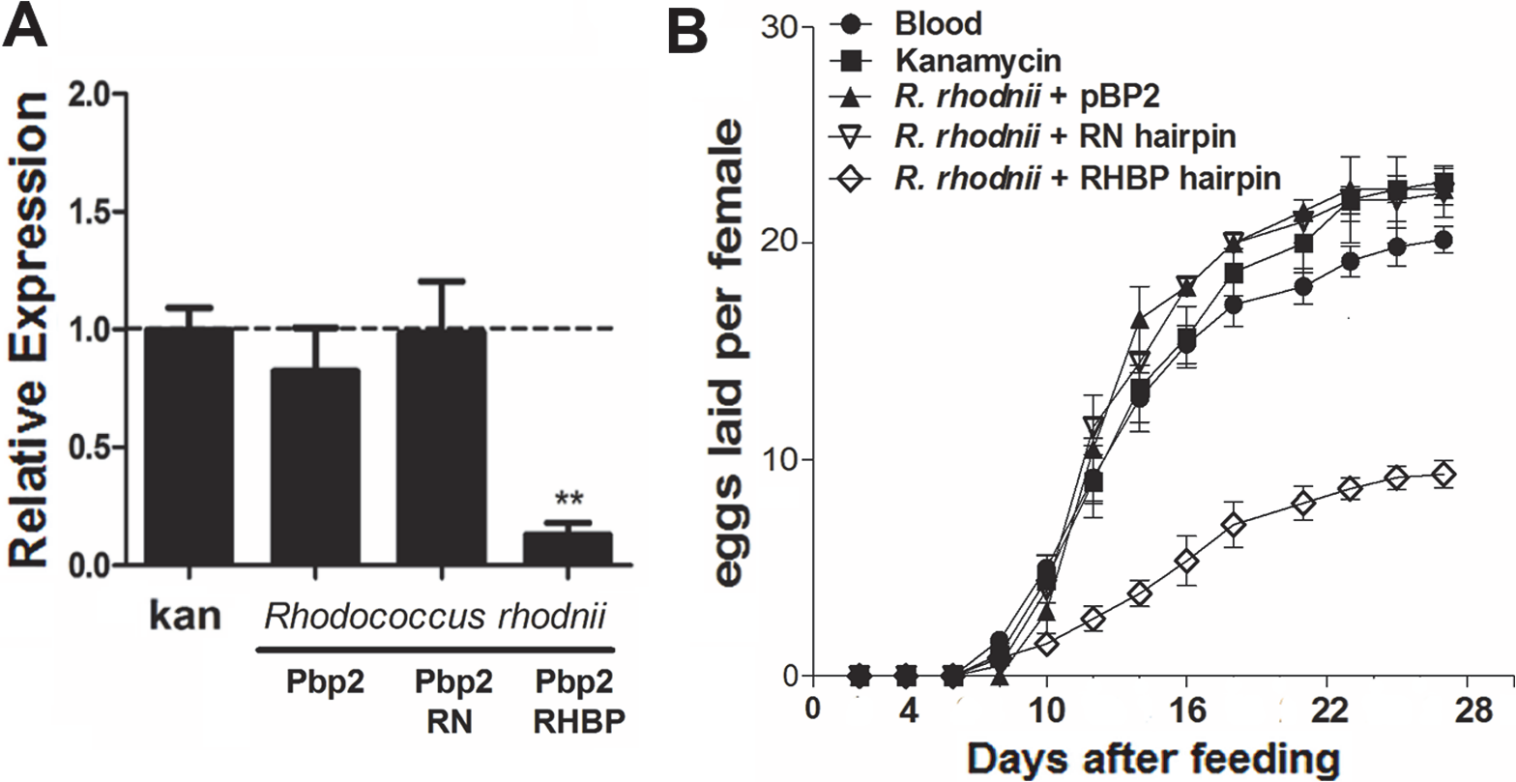
Reduction in RHBP expression and oviposition in females fed with *R. rhodnii* producing a RHBP dsRNA hairpin. (A) Females were fed blood with kanamycin alone (kan) or containing 10^5^ CFU/mL *R. rhodnii* transformed with pBP2lac without insert (pBP2lac), expressing a RHBP dsRNA hairpin (pBP2lacRHBP) or a random nucleotide hairpin (pBP2lacRN). Error bars represent SEM of three biological replicates (n = 12 each). Asterisk indicates statistically different values (*T*-test, *P*< 0.05). (B) Reduction of eggs in one oviposition cycle. Three biological replicates (n = 6 each). Error bars represent SEM. Asterisks denote statistically significant values relative to control fed blood alone (*T* test, *P*<0.05).

We amplified, cloned, sequenced, and expressed a RHBP dsRNA hairpin in the natural symbiont of *R. prolixus, R. rhodnii*, using pBP2lacZ, an integration plasmid with a constitutive expression promoter and a kanamycin resistance gene (SI Materials and Methods). A random nucleotide (RN) hairpin was used as a control. Insects were fed blood containing kanamycin and *R. rhodnii* that expressed these hairpins, bacteria with integrated plasmid without inserts, or blood free of bacteria. We used a concentration of bacteria similar to the normal level in the *R. prolixus* midgut after starvation (10^5^CFU). Inhibition of RHBP mRNA expression was confirmed by q-PCR (Fig. 5A), observing a significant (*T* test, *P*<0.05) reduction (87%) of the expression five days after the feeding. Oviposition was affected in a similar manner as with *E. coli*, obtaining a significant reduction (47%) of the total number of eggs laid (ANOVA with Bonferroni post hoc test, *P*<0.001). No white eggs where observed and the viability of the laid eggs was 100%.

## Discussion

Oral administration of *E. coli* expressing dsRNA for two *R. prolixus* genes involved in oxidative stress achieved systemic gene knockdown in adult and immature stages, with subsequent effects on oviposition and molting. Knockdown was effective in females and third instars, showing effects on expression profiles at least one week after oral delivery of bacteria. Lower concentrations of the natural symbiont of the insect, *R. rhodnii*, achieved similar effects. With both bacterial systems, we show deleterious effects on insect fitness. We propose that the *E. coli* system may be used as an effective high throughput system to screen for targets to be further tested with the symbiont, *R. rhodnii*.

Insects fed *E. coli* expressing RHBP dsRNA recovered to control expression levels ten days after feeding. This recovery is also evident in females fed bacteria during two consecutive oviposition cycles. The oviposition phenotype during the second knockdown event is not affected by the previous knockdown. The oviposition phenotype with *R. rhodnii* suggests that RHBP must have reached normal levels after an initial knockdown. Oral delivery of bacteria expressing dsRNA produces a temporary knockdown that is shorter-lived compared with knockdown generated by intrathoracic injection of one microgram of RHBP dsRNA [30]. Temporary knockdown is also seen after feeding nymphs 1 μg/μL of dsRNA for salivary gland nitrophorins [39]. Although the temporary effects may be construed as a limitation of our system, the estimated dsRNA dose with *E. coli* is 100 times lower compared to oral delivery of pure dsRNA and similar to the intrathoracic inoculation doses, suggesting that bacteria are effective dsRNA vehicles through the oral route. If the target is properly selected, the temporary silencing should produce strong enough phenotypes to affect vector development and/or competence.

Lower symbiont concentrations were needed to induce knockdown compared to *E. coli*. The *E. coli* strain is induced before oral delivery to expresses dsRNA from high copy number plasmids whereas *R. rhodnii* contains a hairpin construct under a constitutive promoter that is integrated into the chromosome. The dsRNA expression kinetics of the symbiont in the midgut remains to be determined. Symbionts are known to replicate in the midgut and are thought to subsequently lyse [23]. Whether dsRNA was released upon bacterial lysis is not known. The pathway by which the dsRNA is taken up from the intestine and delivered to other tissues is not fully understood either. Some insects have transmembrane proteins thought to transport dsRNA [40, 41]. Alternatively, there may be an endocytic pathway [42]. Regardless of the dsRNA release mechanism and uptake route, the silencing mechanism with both *E. coli* and the symbiont had strong physiological effects. Silencing efficacy may be improved by optimizing the symbiont expression system, taking into consideration the dynamics of target gene expression and bacterial promoter activity in the insect midgut.

Catalase expression was reduced in various tissues, suggesting that silencing efficacy is not determined by the tissue proximity to the dsRNA source. Also, it appeared that the earlier the developing stage receives the bacterial treatment, the higher the impact it has on fitness, as first instars presented a 30% mortality rate. Taken together, the data suggest that CAT could be combined with other molecular targets to reduce insect fecundity and development through RNAi. The importance of CAT in parasite survival and insect fecundity has been demonstrated in other vector insects such as *Anopheles gambiae* [43] and *Lutzomyia longipalpis* [28]. Knockdown of CAT in *An. gambiae* resulted in a decrease in *Plasmodium berghei* burden [44] and a decrease of 45% larvae produced by female mosquitoes [43], proving that ROS detoxification by CAT is involved in immunity and protects the ovary and embryo from oxidative damage. In *L. longipalpis* CAT knockdown reduces *Leishmania mexicana* burden [45] as well as female fitness and reproductive capacity [28]. Future studies should determine the effect of silencing CAT on *T. cruzi* development. If CAT silencing affects both, parasite and vector development, this strategy may function to simultaneously reduce transmission and vector colonization.

Given that our proof-of-concept with *E. coli* was efficient at gene knockdown, we propose to use *E. coli* to develop a high throughput screen for novel targets for this strategy and as a tool to study vector physiology. The *E. coli* strain provides a better screening tool because it can be transformed at high efficiencies with extrachromosomal high copy plasmids and it grows more quickly. In contrast, the *R. rhodnii* transformation system requires random integration into one of several potential chromosomal attachment sites. This makes it more difficult to select for transformants that have the same genetic background for screening purposes. Molecules identified through *E. coli* screening can be selected for integration into specific attachment sites in the *R. rhodnii* chromosome for further experiments. The *E. coli* system may be used in future studies as a research tool to identify target genes that produce higher nymph or egg mortality levels. It may also be used to evaluate the effect of silencing triatomine genes on *T. cruzi* interactions with the vector and potential transmission-blocking effects that may complement the control strategy.

Selection of potential molecular targets should consider the kinetics of gene expression after feeding and the half-life of the protein being silenced. An ideal target in the development of a symbiont-based control strategy with *R. rhodnii* should be expressed maximally one week after the blood meal to take advantage of the bacterial replication cycle in the midgut [23]. In addition, the protein being knocked down should have a high turnover rate, as the silencing effect may be overshadowed by the presence of previously produced protein. The availability of the *R. prolixus* genome and tissue-specific cDNA sequences will greatly facilitate target screening for this strategy.

We showed for the first time that transformed symbionts fed in blood are effective at delivering dsRNA and silencing genes in a hemipteran disease vector. Future studies will evaluate silencing efficacy after delivery in a paste-like formulation to simulate natural coprophagous transmission [16]. The bacterial integration system we used is stable even in the absence of antibiotic selection once introduced into the vector [46]. The availability of effective systems for symbiont delivery through simulated feces and stable transformation make it feasible to produce gene-silencing microbiota. Horizontal transmission among exposed insects can be a sustainable alternative to traditional insecticides. This could reduce vector competence and development, with low ecological impact due to target specificity [10]. The selection of resistance in natural populations may be minimized if multiple genes are simultaneously targeted, similar to what occurs with *Bacillus thuringiensis* in mosquitoes, where Cry and Cyt toxins synergize with each other and avoid resistance [47]. The production of bacteria that express dsRNA for several molecular targets simultaneously and constitutively would be recommended [48]. The future applications of the genetic modification of insect microbiota to produce RNAi are wide. These post-genomic tools for the study of insect physiology, vector-parasite interactions and ultimately control strategies may be applied to other insects of importance to human and animal health.

## Supporting Information

S1 Text Supporting information for materials and methods describing insects, plasmids and bacterial strains, dsRNA purification, feeding conditions, verification of knockdown by q-PCR, phenotype description, evaluation of RHBP levels in hemolymph, catalase activity, egg fertilization evaluation through detection of male-specific genes and determination of reactive oxygen species in the female midgut. S1 Table in S1 Text. Mortality and molting rates of first and third instar nymphs of *R. prolixus* fed with *E. coli* expressing dsRNA for RHBP or CAT.
(PDF)

S1 Fig Effects of bacteria in the blood on the feeding and digestion processes in *R. prolixus* females. (TIF)

S2 Fig Effects of the repeated ingestion of 5.4 × 10ˆ7 cfu/ml *E. coli* HT155(D3) expressing dsRNA RHBP on oviposition of *R. prolixus* adult females. (TIF)

S3 Fig Double-stranded RNA of the RHBP and CAT genes of *R. prolixus* and ANT gene from *A. thaliana* produced using an in vivo expression in bacteria *E. coli* strain HT115. (TIF)

S4 Fig Identification of a male specific gene for fertilization confirmation by PCR. (TIF)
