# Supplementary material for: Genetically Modifying the Insect Gut Microbiota to Control Chagas Disease Vectors through Systemic RNAi
Source: PLoS Negl Trop Dis. 2015 Feb 12;9(2):e0003358. doi: 10.1371/journal.pntd.0003358 (PMC4326462; doi:10.1371/journal.pntd.0003358)
Supplement: S1 Text — S1 Table in S1 Text. Mortality and molting rates of first and third instar nymphs of R. prolixus fed with E. coli expressing dsRNA for RHBP or CAT. (PDF) [file pntd.0003358.s001.pdf]

Insects

Insects were taken from a colony of *R. prolixus* maintained at 28°C and 70% relative humidity. First instar nymphs were synchronized for feeding by eclosion date. Third instar nymphs were synchronized after feeding second instars. After molting, adult females were maintained with males and fed onceon a rabbitto synchronize their physiological state. Three weeks later, experimental females or third instar nymphs were offered a blood meal through an artificial membrane feeder [1] containing fresh rabbit blood collected from the auricular vein under approved animal care and use protocols.

Plasmids and bacterial strains.

The RHBP gene (GenBank: AF493801.1) was cloned from cDNA generated from 3rd instar nymphs (carcass) and the catalase gene from cDNA generated from adult females (anterior and posterior midgut, hindgut, fat body, ovaries, heart and one fourth of the thorax cuticle). The extraction was performed with TRIzol reagent according to manufacturer's directions (Invitrogen, CA, USA). The RNA for RHBP was resuspended in DEPC water, 1 µg was treated with DNAseI (Promega, MI, USA) and cDNA synthesized with MgdT primer and M-MLV reverse transcriptase (Promega, MI, USA) [2]. A PCR product was generated with the primers RHBPF1 5´AAACTGTTCGGTTATCTAATCA 3´ and RHBPR1 5´CAACTGTACGCTTGGTACG3´ in a 25 µl PCR reaction that included 3 mM $MgSO_4$, 0.5 µM of each primer, 1.0 U Taq Platinum Taq DNA polymerase High Fidelity (Invitrogen), 60 mM Tris-SO4 (pH 8.9), 18 mM Ammonium sulfate, 20 µM each dNTP and 1 µl cDNA. The amplification was performed in an Eppendorf Mastercycler (Hamburg-Eppendorff, Germany) with an initial denaturation at 94°C for 1.5 min, followed

by a touchdown procedure of 9 cycles of 94°C for 30 sec with a gradual decrease of the annealing temperature from 55°C to 44°C for 30 sec and a 1 min extension at 68°C. A final amplification was performed with 30 cycles of 94°C for 30 sec, 43°C for 30 sec and 1 min extension at 68°C. The RHBP PCR product was cloned in the pGEM-TEasy vector (Promega, Madison, WI, USA). A construct with the T7 promoter flanking RHBP was generated from this plasmid by performing a second PCR under the same conditions, using the following primers: T7rhbpF 5´GTAATACGACTCACTATAGGGCGAATTGAA AACTGTTCGGTTATCTAATC 3´ and T7rhbpR 5´GTAATACGACTCACTATAGGG CGAATTGTCAACTGTACGCTTGGTA 3´. With the primers RHBP112F 5´GATCCGATAGGGCACGCCAATTAGGAATTCCTAATTGGCGTGCCCTATCTTTT TTTTA (58nt) and RHBP112R 5´AGCTTAAAAAAAAGATAGGGCACGCCAATTAGG AATTCCTAATTGGCGTGCCCTATCG (58nt) a RHBP hairpin fragment and a random nucleotide control (RHBPNCF 5´GATCCATGCACCATGACCATCTAGGAATTCCTAG ATGGTCATGGTGCATTTTTTTTTA, RHBPNCR5´AGCTTAAAAAAAAATGCACCA TGACCATCTAGGAATTCCTAGATGGTCATGGTGCATG) were cloned into a pBP2 plasmid.

The RNA for CAT was resuspended in DEPC water, 1 µg was treated with DNAse I (Fermentas International Inc., Burlington, Canada) and cDNA synthesized with random primers and the High-Capacity cDNA Reverse Transcription Kit (Applied Biosystems, Foster City, EUA). A PCR product was generated with the primers Cat_RNAi_F 5´TAATACGACTCACTATAGGGGGAGCGTTCGGTTACTTTGA3´ and Cat_RNAi_R 5´TAATACGACTCACTATAGGGGCAAGTTTCACCTCGGTCAT 3´ in a 15 µl reaction

including 5µL Taq DNA Polymerase buffer with 100 mM Tris-HCl (pH 8.8 at 25°C) and 500 mMKCl, 3µl of 25mM $MgCl_2$, 1µl of 10mMdNTPs, 0.4 µl of each primer (15µM), 0.2µl of Taq DNA Polymerase (5U/µl, Fermentas International Inc., Burlington, Canada), and 1 µl of cDNA. The amplification was performed in an Eppendorf Mastercycle personal thermalcycler with an initial denaturation at 94°C for 3 min, followed by 30 cycles of 94°C for 30 sec, 55°C for 30 sec and 30 sec extension at 72°C; with a final 4 min extension at 72°C.

As a control for silencing specificity, dsRNA of an *Arabidopsis thaliana* gene ANT (GenBank: U41339.1), an APETALA2-like gene with pleiotropic roles in ovule development and floral organ growth,was produced as described [3] using the primers that include the T7 promoter, ArabidT7f 5´ GTA ATA CGA CTC ACT ATA GGG CGA ATT GGG TGG AGG ATT TCT TTG GGA CC 3´ and ArabidT7r 5´ GTA ATA CGA CTC ACT ATA GGG CGA ATT GAC GCC TCG GTA TTG AGA AGT TCG 3´.

All PCR products were cloned with the pGEM-T easy kit (Promega, Madison, WI, USA). Electroporation was performed with the BioRadGenePulser II (Hercules, CA, USA). The PCR products were ligated into pGEM-T Easy (Promega, Madison WI) and cloned into *E. coli* XL-1 Blue cells (RHBP and ANT) and into *E. coli* DH10b (CAT). The plasmids were purified and cloned into *E. coli*HT115(DE3) competent cells as described by Timmons [4]. Bacterial culture for dsRNA production.

A single colony of the *E. coli* HT115(DE3) containing the pGEM-T plasmid with inserts for RHBP, CAT, and the ANT gene, and without plasmid was grown in 3 ml LB media containing ampicillin (100 µg/ml) and/or tetracycline (12.5 µg/ml) overnight at 37°C and

180 rpm. The bacterial starter culture was diluted 100 fold in 50 mL of 2xYT media containing 0.4 mM IPTG and ampicillin (100 µg/ml) and incubated 4-5 hrs at 37°C and 180 rpm until the OD_{600} reached ~1.0. Initially, a culture without IPTG was prepared as control for knockdown specificity related to induction of dsRNA synthesis. The bacterial cells were harvested by centrifugation at 4,400 *g* for 10 min at 4°C. A pellet from 20, 24, 32 or 40 ml of culture was resuspended in 4 mL of fresh rabbit blood for artificial feeding.

A single colony of the *R. rhodnii* containing the RHBP-pBP2 plasmid with inserts for RHBP and the negative control hairpins, and without plasmid was grown in 3 ml BHI media containing kanamicin (100 µg/ml),for 72 hours at 28°C and 180 rpm. The bacterial starter culture was diluted 100 fold in 50 mL of BHI media and incubated 24hrs at 28°C and 180 rpm. The bacterial cells were harvested by centrifugation at 4,400 *g* for 10 min at 4°C. A pellet of cells was resuspended in 4 mL of fresh rabbit blood for artificial feeding.

dsRNA purification

The dsRNA was purified as described by Ongvarrasopone [5]. Briefly, 1mL of the induced 2xYT culture of *E. coli* HT115(DE3) was grown until the OD_{600} reached 1. The bacterial cells were harvested by centrifugation at 3700 *g* for 10 min at 4°C. The cell pellet was resuspended in 50 µl of 0.1% SDS and lysed by boiling 2 min. 65 µl of RNaseA buffer (300mM sodium acetate, 10 mM Tris-Cl pH 7.5 and 5 mM EDTA) was added with 1 µg of RNase A (Sigma Aldrich, St Louis, USA). After incubation at 37°C for 5 min to remove single stranded RNA,the dsRNA was purified with 500 µl of Trizol reagent (Invitrogen,

Carlsbad CA) per manufacturer´s directions. The dsRNA concentration was estimated on a Nanodrop 1000 Spectrophotometer v.3.7 (Thermo Fisher Scientific, Waltham, USA).

Feeding conditions

Each group was fed separately on individual feeding chambers containing blood with: *E. coli* expressing dsRNA for RHBP, CAT, ANT or bacteria expressing no dsRNA (without vector). An additional group without bacteria was used as a normal feeding control. For time-dependent experiments, insects were chosen randomly from the group and dissected at each time point (days one, three, five, seven or ten after feeding). For oviposition experiments, insects were maintained in individual flasks and eggs were collected daily. Fresh feces and inoculated blood were cultured in LB agar with ampicillin (100 µg/mL) and/or tetracycline (12.5 µg/mL) to obtain bacterial counts.

First stage nymphs, obtained from eggs laid on the same day, were separated into groups of twenty, and fed simultaneously at weekly intervals to synchronize molting. Synchronized third instar nymphs were randomly assigned to groups of five animals per treatment and molting was evaluated daily. Gene expression was evaluated in pools of two individuals by q-PCR as described below. The effect of bacteria on blood ingestion during oral delivery was evaluated by weighing groups of nine females, in three biological replicates, before and after feeding blood with and without bacteria. Individuals that did not ingest blood based on weight change were excluded from all experiments. For bacterial dose optimization and quantification of the mRNA levels by q-PCR, six insects were used for the 2.24 x 10^7cfu/ml dose, 12 insects for the 3.35 x 10^7cfu/ml dose, and eight insects for the 5.4 x 10^7cfu/ml dose. All subsequent q-PCR experiments with RHBP dsRNA were

performed with six individuals per replicate. After standardization of feeding conditions with RHBP, CAT groups were fed under the same conditions using four insects per replicate, using individual insects to evaluate gene expression, catalase activity and determination of reactive oxygen species (ROS).

Verification of knockdown by q-PCR

The RNA was collected from fat bodies, ovaries, posterior- and anterior-midguts of individual females at days three, five, seven and ten after feeding for RHBP experiments and days one, three and five after feeding for CAT experiments. All samples were purified using TRIzol reagent (Invitrogen), and resuspended in 20 µl of DEPC water. One microgram of total RNA was treated with DNAse I and reverse transcribed with random primers using the High Capacity cDNA Reverse Transcription Kit (Applied Biosystems, CA, USA), per manufacturer's instructions, in a final volume of 20 µl. A 1:10 dilution of the final cDNA product was used. q-PCR was performed using the following primers: Real TimeRHBPF 5' - TCC TTC ACA CTC TCC GCA AC - 3' (foward) and Real TimeRHBPR 5' - GTA CGC TTG GTA CGC CAC TT - 3' (reverse), for the RHBP gene; and RpActR1TF 5' - CCA TGT ACC CAG GTA TTG CT - 3' (foward), and RpActR1TR 5' - ATC TGT TGG AAG GTG GAC AG - 3' (reverse) for the actin gene, used as internal control in RHBP experiments; Real TimeCatF 5' –TTCATCCACACGCAGAAGAG - 3' (foward) and Real TimeCatR 5' –GCAAGTTTCACCTCGGTCAT - 3' (reverse), for the CAT gene; and RNAr18sF 5' –TGTCGGTGTAACTGGCATGT - 3' (foward), and RNAr18sR 5' –TCGGCCAACAAAAGTACACA - 3' (reverse) forthe RNAr18S geneas internal control in CAT experiments [6]. Each primer was added at 600 nM to 7.5 µl of

Power SYBR green PCR master mix (Applied Biosystems) and 5 µl of cDNA for a final volume of 15 µl. The PCR was initiated with a hot start of 95°C for 10 min, followed by 40 cycles of 15 sec at 95°C for denaturation and 60 sec at 60°C for annealing and extension; plus a standard melting curve in a StepOne real time thermalcycler (Applied Biosystems, Carlsbad, CA, USA).

Phenotype description

First and third stage nymphs were maintained in groups of 15 and five, respectively, and observed daily to track mortality and molting. Females were maintained in individual chambers during the oviposition cycle and eggs were collected daily. Ovaries were dissected and photographed witha Feldmann Wild-Leitz (FWL-DIGI-PRO 5.0M cm) microscope with Micrometrics SE Premium software. Eggs were maintained in separate flasks, separated by individual and oviposition day, and monitored until they hatched.

Evaluation of RHBP levels in hemolymph

Hemolymph was collected from the severed femur of each female (at least three per experimental treatment, in three separate biological replicates) and immediately 10 µl were mixed with a phenylthiourea crystal and one volume of protease inhibitors cocktail (0.05 mg/mL antipain, 0.05 mg/mL Soybean trypsin inhibitor, 0.05mg/mL Lima bean trypsin inhibitor, 1mM benzamidine and 0.05 mg/mlleupeptin). SDS-PAGE was performed to assess the integrity of the protein profile of each hemolymph sample. A volume equivalent to 40 µg of total protein, based on sample absorbance at 230 nm, was mixed with SDS-PAGE sample buffer (0.5M Tris-HCl pH 6.8, 4.4% SDS, 300mM mercaptoethanol, 10

mg/ml bromophenol blue, final concentration). Electrophoresis in a 15% polyacrilamide gel (acrylamide/bis-acrylamide 29:1), using the buffer system described for Laemmli [7] with a constant voltage of 70 mV was performed in a Bio-Rad MiniProtean II gel system (Hercules, CA, USA). Each gel was stained with Coomassie Blue and destained with a mixture of water:ethanol:acetone (40:40:20). Molecular weight was determined with a Spectra™ Multicolor Broad Range Protein Ladder (FermentasBurlington, ON,Canada). Binding of heme to protein from hemolymph was measured by progressively adding hemeto 1 µl female hemolymph and recording the absorption spectra after each addition (0.1 nmol) of 0.1 mM hemin prepared in 0.1 N NaOH [8], using a UV 2550 spectrophotometer (Shimadzu, Japan) with a UVProbe software.

Catalase activity

Midguts from insects fed *E. coli* expressing dsRNA CAT and the controls (3/group, two replicates) were individually homogenized in 120 µl of PBS with 5 µl of the same protease inhibitor cocktail used above. Catalase activity was measured spectrophotometrically by normalizing the homogenate protein content, diluting 40 µl in860 µl of PBS and adding 100 µl of 9mM hydrogen peroxide. Absorbance was measured at 240 nm for 1 min at room temperature [9]. The same procedure was performed adding 2 µl of amino-triazol(1.0 mM) as a specific inhibitor of catalase.

Egg fertilization evaluation through detection of male-specific genes.

Pools of three eggs (seven days old) per female, of five females per group, were analyzed. Eggs, viable and non viable, from the CAT silenced animals were analyzed separately.

Sampleswere homogenized with lysis buffer (10mM Tris pH 8, 0.1 M EDTA pH 8, 0.5% (w/v) SDS and 1µg/µlRNAse) and incubated for 10 min at room temperature. Proteinase K was added to a final concentration of 20 µg/ml and incubated at 50°C for 3 h. DNA was extracted by mixing and incubating 10 min with one volume of Tris-saturated phenol, pH 8. The extraction was repeated twice by centrifugation at 5,000 g for 15 minutes at room temperature and the supernatant was precipitated by addition of 0.2 vol of 10M ammonium acetate and 2 volumes of 100%ethanol. The pellet was washed twice at 5,000 g in 30µl of DEPC water. PCR was performed with the primers for the Rp_1400 gene of the Y chromosome (GeneBank: JX559072): Rp_1400F -TCCTCCGCCTTGCTTCTCTGT - and Rp_1400R - GTGCGGGCGGTGGATTG -, using a program of 3 min at 95°C, 35 cycles of 95°C per 30 sec, 55°C per 30 sec and 72°C per 1 min; and a final 7 min extension at 72°C. Products were visualizedby ethidium bromide staining of 1.5% agarose gels after electrophoresis at 5 V/cm in 1X TAE.

Determination of reactive oxygen species in the female midgut

Midguts were incubated with a 50 µM solution of the oxidant-sensitive fluorophoredihydroethidium (DHE) (Invitrogen). After a 20 min incubation at room temperature, the midguts were washed in 0.15M NaCl and transferred to a glass slide for epifluorescence microscopy (Zeiss Observer.Z1 with Zeiss Axio Cam MrM) using a Zeiss-15 filter set (excitation BP 546/12; beam splitter FT 580; emission LP 590). Comparison of fluorescence levels among distinct micrographs was performed under identical conditions, using the 20x objective and 100 ms exposure time in each experiment. To evaluate the

effects of the presence of bacteria on the amount of blood ingested, and R ratio was calculated [10] as the weight of blood ingested (g)/initial weight of the insect(g).

References

1. Garcia E, Garcia L, Macarini J, Uubatuba F (1975) Alimentação de *Rhodnius prolixus* no laboratório. A Ac Br Ciên 47: 537-545.
2. Ursic-Bedoya RJ, Nazzari H, Cooper D, Triana O, Wolff M, et al. (2008) Identification and characterization of two novel lysozymes from *Rhodnius prolixus*, a vector of Chagas disease. J Insect Physiol 54: 593-603.
3. Cooper DM, Chamberlain CM, Lowenberger C (2009) Aedes FADD: a novel death domain-containing protein required for antibacterial immunity in the yellow fever mosquito, *Aedes aegypti*. Insect Biochem Mol Biol 39: 47-54.
4. Timmons L, Tabara H, Mello CC, Fire AZ (2003) Inducible systemic RNA silencing in *Caenorhabditis elegans*. Mol Biol Cell 14: 2972-2983.
5. Ongvarrasopone C, Roshorm Y, Panyim S (2007) A simple and cost effective method to generate dsRNA for RNAi studies in invertebrates. Sci Asia 33: 35-39.
6. Majerowicz D, Alves-Bezerra M, Logullo R, Fonseca-de-Souza AL, Meyer-Fernandes JR, et al. (2011) Looking for reference genes for real-time quantitative PCR experiments in *Rhodnius prolixus* (Hemiptera: Reduviidae). Insect Molec Biol 20: 713-722.

7. Laemmli UK (1970) Cleavage of structural proteins during the assembly of the head of bacteriophage T4. Nat 227: 680-685.

8. Oliveira PL, Kawooya JK, Ribeiro JMC, Meyer T, Poorman R, et al. (1995) A Heme-binding protein from hemolymph and oocytes of the blood-sucking insect, *Rhodnius prolixus*. J Biol Chem 270: 10897-10901.

9. Aebi H (1984) Catalase in vitro. Meth Enzymol 105: 121-126.

10. Davey K (2007) The interaction of feeding and mating in the hormonal control of egg production in *Rhodnius prolixus*. J Insect Physiol 53: 208-215.

Table S1. Mortality and molting rates of first and third instar nymphs of *R. prolixus* fed with *E. coli* expressing dsRNA for RHBP or CAT.

Group	1st instar nymphs				3rd instar nymphs			
	N	Replicates	Mortality rate	Molting rate	N	Replicates	Mortality rate	Molting rate
Bacteria with RHBP dsRNA	15	2	30%	0%	5	2	0%	20%
Bacteria with CAT dsRNA	14	2	32.1%	0%	5	2	0%	20%
Bacteria with ANT dsRNA	12	2	0%	100%	5	2	0%	100%
Bacteria without dsRNA	15	2	8.33%	100%	5	2	0%	100%
Without bacteria	15	2	0%	93.3%	5	2	0%	100%

‡ First instars were fed 2.5 x 10^7 CFU/mL blood, third instars 5.54 x 10^7 CFU/mL blood .
