# Supplementary figures and images for: Genetically Modifying the Insect Gut Microbiota to Control Chagas Disease Vectors through Systemic RNAi
Source: PLoS Negl Trop Dis. 2015 Feb 12;9(2):e0003358. doi: 10.1371/journal.pntd.0003358 (PMC4326462; doi:10.1371/journal.pntd.0003358)

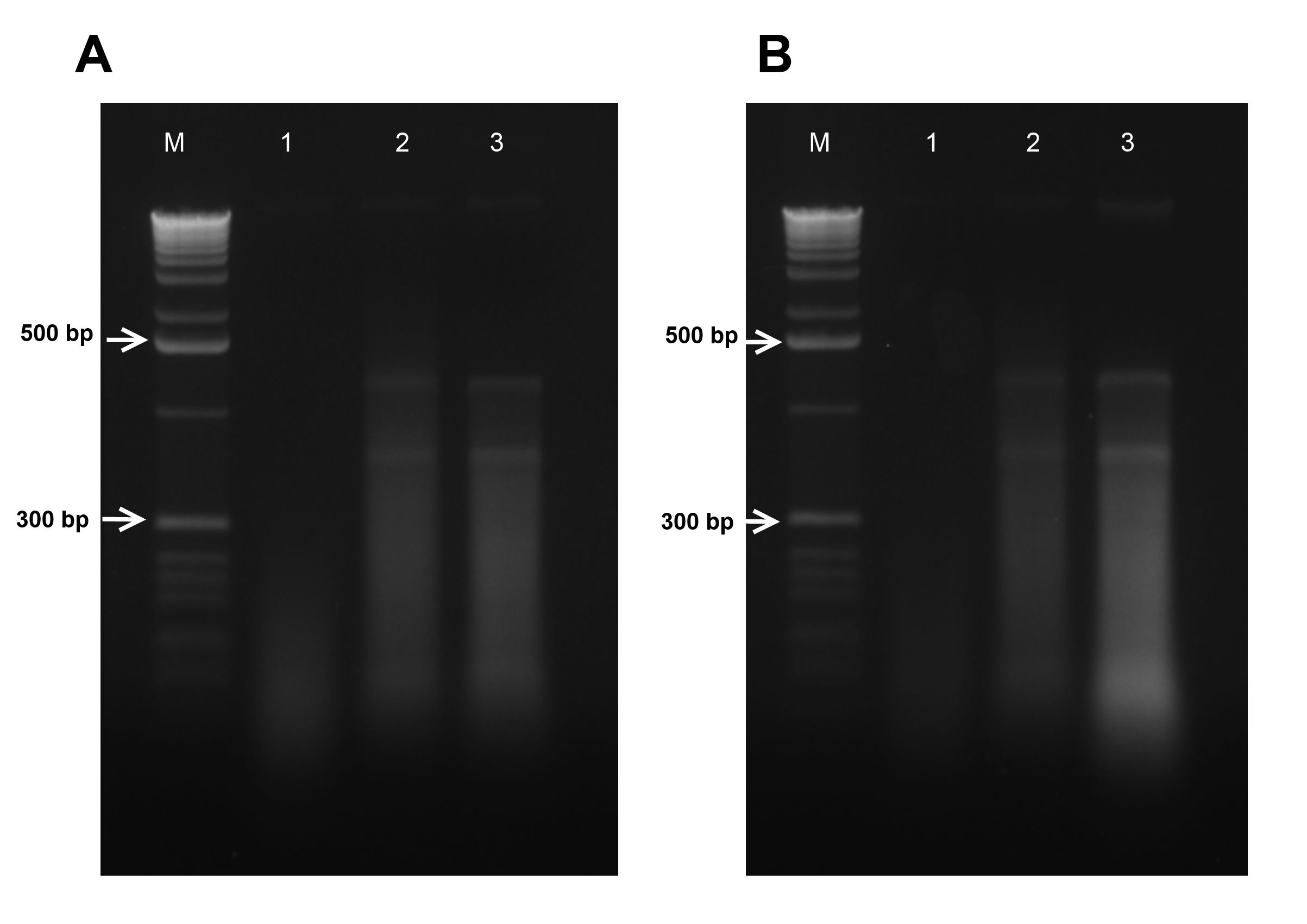

Supplement: S1 Fig — (TIF) [file pntd.0003358.s002.tif]

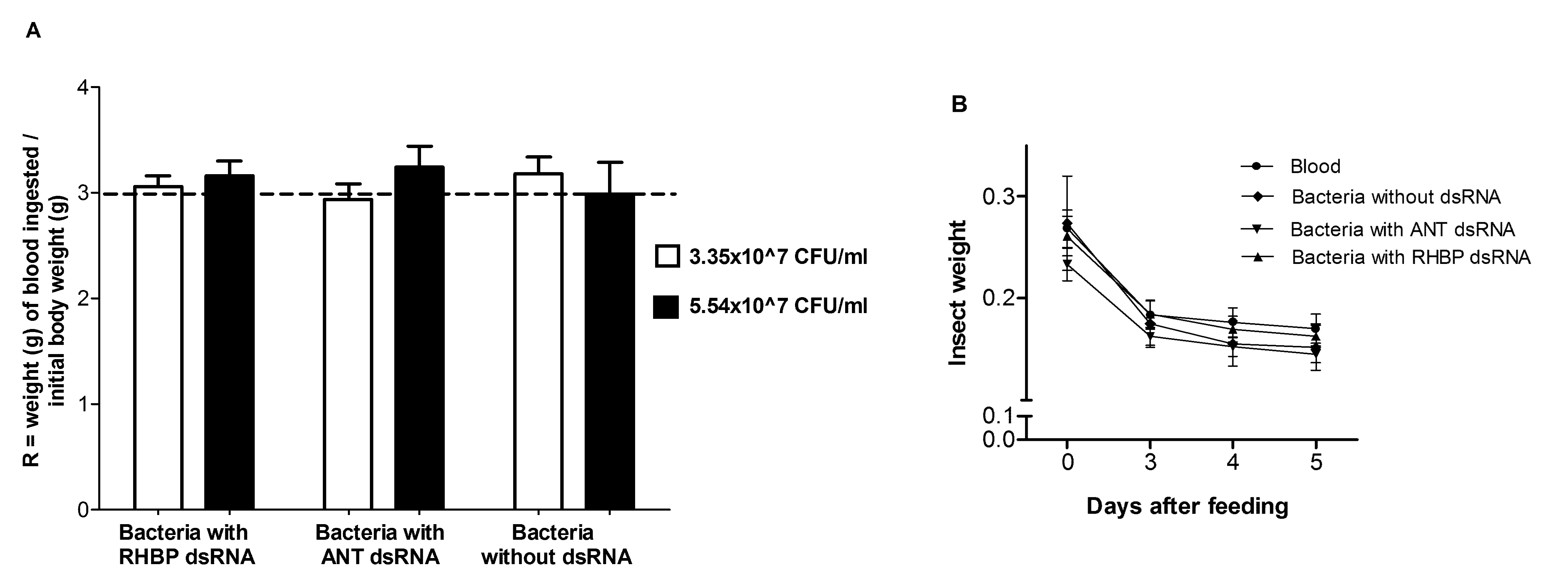

Supplement: S2 Fig — (TIF) [file pntd.0003358.s003.tif]

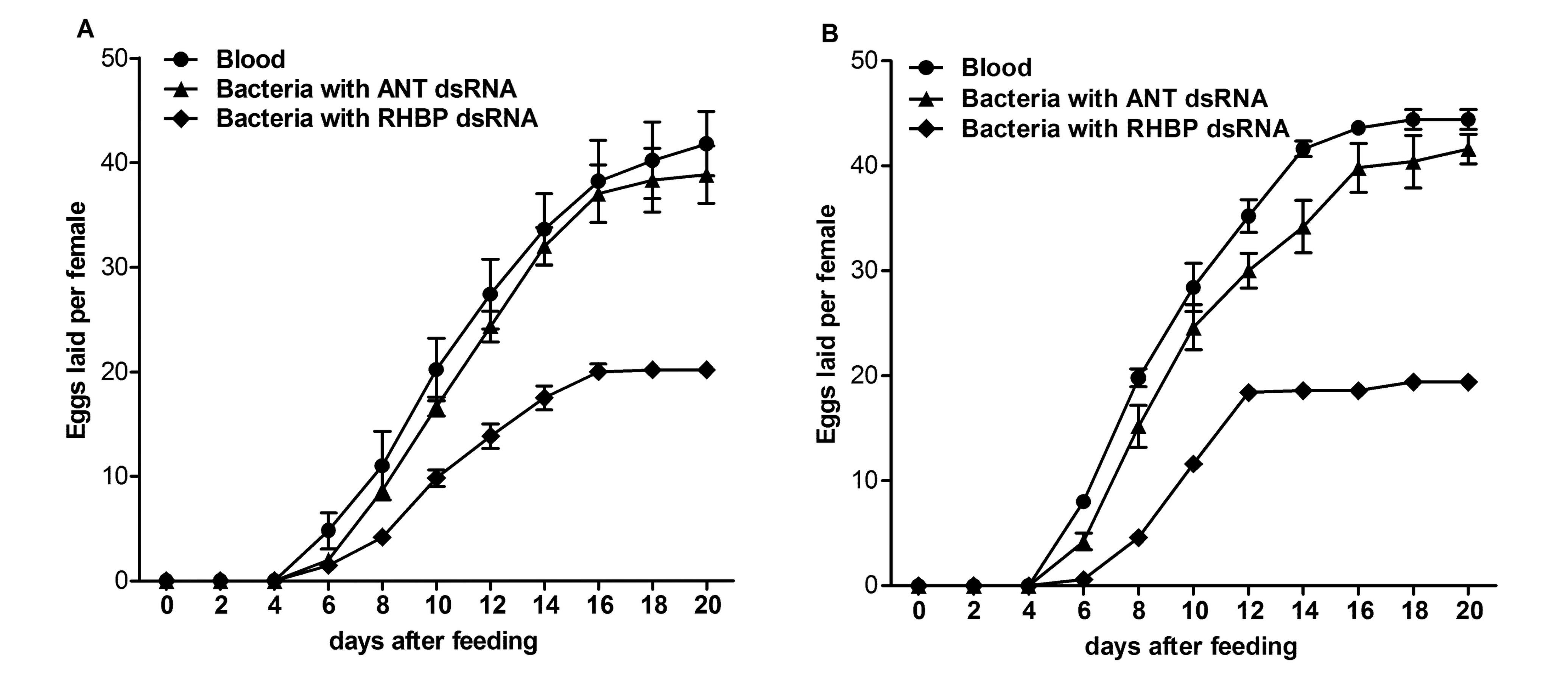

Supplement: S3 Fig — (TIF) [file pntd.0003358.s004.tif]

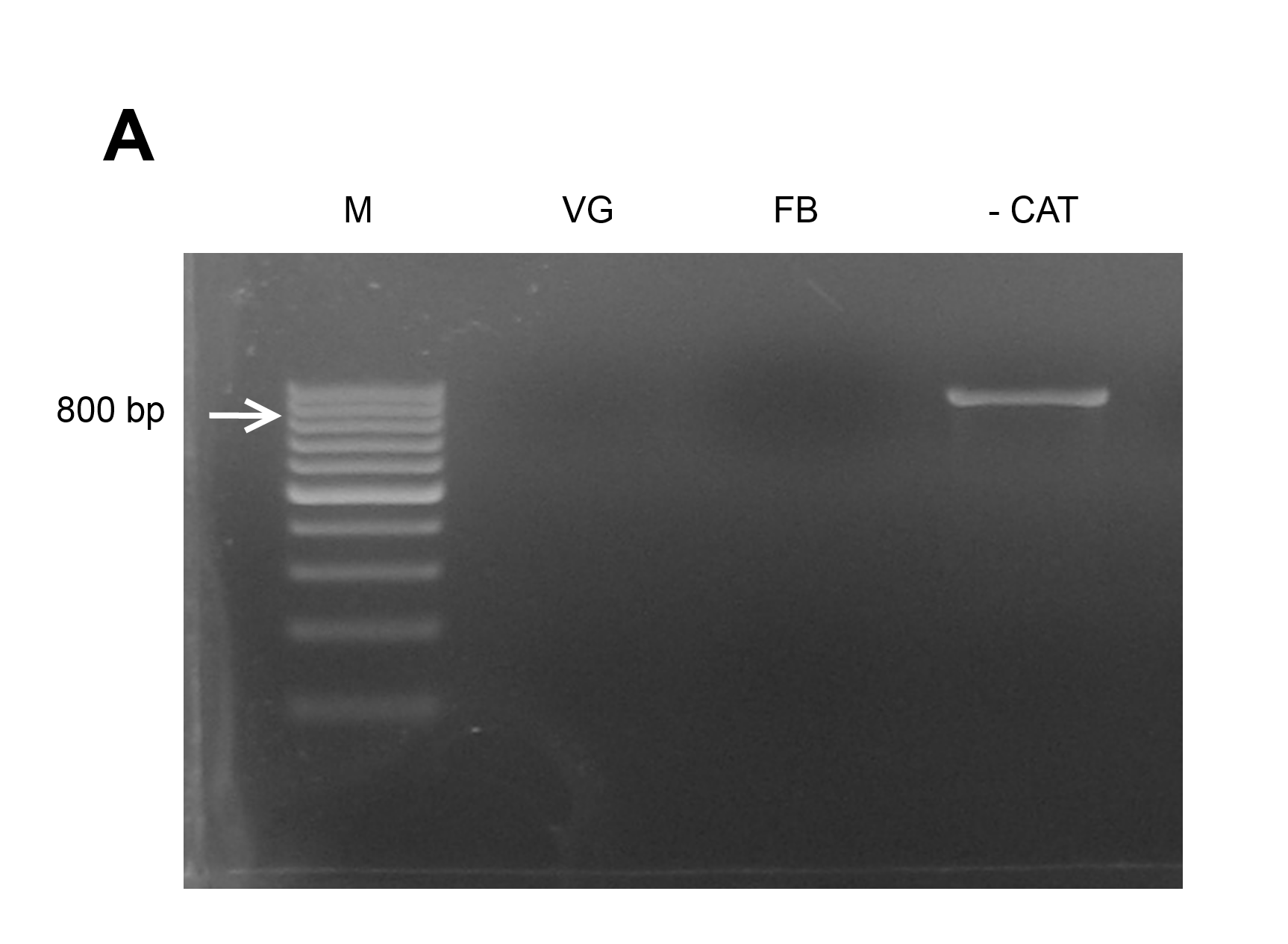

Supplement: S4 Fig — (TIF) [file pntd.0003358.s005.tif]
